# Female Burying Beetles Benefit from Male Desertion: Sexual Conflict and Counter-Adaptation over Parental Investment

**DOI:** 10.1371/journal.pone.0031713

**Published:** 2012-02-15

**Authors:** Giuseppe Boncoraglio, Rebecca M. Kilner

**Affiliations:** Department of Zoology, University of Cambridge, Cambridge, United Kingdom; University of California Santa Barbara, United States of America

## Abstract

Sexual conflict drives the coevolution of sexually antagonistic traits, such that an adaptation in one sex selects an opposing coevolutionary response from the other. Although many adaptations and counteradaptations have been identified in sexual conflict over mating interactions, few are known for sexual conflict over parental investment. Here we investigate a possible coevolutionary sequence triggered by mate desertion in the burying beetle *Nicrophorus vespilloides*, where males commonly leave before their offspring reach independence. Rather than suffer fitness costs as a consequence, our data suggest that females rely on the male's absence to recoup some of the costs of larval care, presumably because they are then free to feed themselves on the carcass employed for breeding. Consequently, forcing males to stay until the larvae disperse reduces components of female fitness to a greater extent than caring for young singlehandedly. Therefore we suggest that females may have co-evolved to anticipate desertion by their partners so that they now benefit from the male's absence.

## Introduction

Sexual conflict arises when selection on costly reproductive traits acts in opposing directions on males and females, thus generating contrasting optima between the sexes (reviewed by [1]). It selects diverse manipulative and harmful behaviours carried out when partners interact at reproduction [1], [2]. Mate desertion, in which one sex abandons the current breeding attempt in favour of future reproduction, leaving the partner to provide costly care for dependent young, is a classic example of sexual conflict over provision of parental investment [1], [3]. The sexes are conventionally viewed as racing to desert first, to avoid paying the costs associated with continued care (*e.g.* [4]). The sex left with the progeny thus loses this form of sexual conflict (*e.g.* [5]).

One key consequence of sexual conflict is that it drives the coevolution of sexually antagonistic traits [1], [2], [6], [7]. Several different coevolutionary scenarios, and their associated terminology, have been defined by Lessells [2]. These specifically spell out potential sequences of adaptations and counter-adaptations by males and females. For example, a shift towards the optimal trait value for one sex might provoke a counter-adaptation in the partner, dragging the trait back towards the partner's optimum [1], [2]. Alternatively, counter-adaptation may lead to an outcome that benefits both sexes equally (*i.e.* a joint optimum or ‘cooperative adaptation’ *sensu* [2]) or it may initiate sexual conflict over a completely new trait (‘adaptation’ [2]). Although studies of sexual conflict over mating have analysed this sort of coevolutionary sequence extensively, it has been relatively little explored when considering sexual conflict over parental investment, where the most typical approach instead is to search for individual strategies that result in evolutionary equilibrium between the sexes [2].

Here we consider how female burying beetles (*Nicrophorus vespilloides*) might have adapted (or counter-adapted) to brood desertion by their mate. Burying beetles reproduce on small vertebrate carcasses [8] and in nature, approximately 85% of *N. vespilloides* broods are tended by at least one male and one female [9] for about 8 days after hatching, whereupon the larvae disperse from the carcass to pupate. When males stay to assist with post-hatching care, they usually leave the brood 2 to 5 days earlier than females [9], [10], presumably because they have greater residual reproductive value all else being equal [11], and because they commonly sire offspring by mating away from the breeding carcass [9]. Males play a key role in defending the carcass from takeover by infanticidal conspecifics or heterospecifics in the early stages of reproduction, but the threat from rivals decreases substantially as larval development progresses becae the value of the carcass declines as it is consumed [12]. The provision of care incurs substantial fitness costs for both sexes [11], [13]. Larvae rely on parental provisioning especially during the first 24 hours, becoming more effective self-feeders thereafter (*e.g.* [14]). Previous work suggests that maternal larval provisioning rates are maximal whether or not the male is present [15], while contribution by males appears not to improve larval fitness, either under laboratory conditions [*e.g.* 14], [15] or in the field [16], [17].

To investigate the potential fitness costs of mate desertion for females, we experimentally simulate male desertion shortly before hatching and ask two related questions: 1) How do the costs associated with uniparental care compare with those incurred under biparental care? The conventional view predicts that females should suffer greater costs when abandoned than when raising offspring with a male (see also [18]). 2) When females are left to care for their offspring singlehandedly, at which stage of larval development are the greatest fitness costs incurred? We predict the costs of parenting should be greatest in the hours immediately after hatching [15] and should continue to accrue at a slower rate thereafter. For both questions, we assessed the fitness costs to females in terms of fecundity in the subsequent breeding bout and individual lifespan. Burying beetles are opportunistic breeders and lifetime fecundity has been previously shown to strongly depend on the number of breeding bouts accomplished during a lifetime [11], [19]. A longer lifespan buys the opportunity for a greater number of breeding bouts. Thus, in this species, lifespan is expected to be a significant component of lifetime reproductive success.

## Materials and Methods

### Ethics statement

This study complies with EU and UK laws for laboratory animal research as authorized by Marie Curie Intra-European Fellowship PIEF-GA-2009-252120. No specific laboratory authorizations or permissions for collection of individuals in the field were required for the described study, as the location for collection was not privately-owned or protected in any way, nor the study involved an endangered or protected invertebrate species.

### *N. vespilloides* colony and housing conditions

We used burying beetles from a captive colony established in 2005 at Cambridge University. The colony is supplemented every year with wild caught beetles from a wooded area near Cambridge, UK. Adults are housed alone in plastic boxes (12×8×2 cm) filled with moist soil, fed twice a week with ca. 0.8 g minced beef and kept at a constant temperature of 21°C and 16 h: 8 h light: dark cycle. For breeding, unrelated pairs are placed into plastic boxes (17×12×6 cm) half filled with moist soil, provided with a freshly thawed mouse carcass (21.94±0.33 SE g, range 15–35 g) and kept in the dark. Larvae disperse from the carcass ca. 8 days later. Sexual maturity is reached ca. 5 weeks after dispersal.

### Experimental protocol

All treatments were run concurrently between January and May 2011. To address question 1), we provided two-three week old virgin females with an unrelated virgin partner and a mouse carcass to start breeding under standard conditions. We simulated mate desertion by removing males from breeding boxes ca. 53 h later, after egg laying and carcass preparation but before hatching, which typically starts 71.28 h±1.47 SE after start of breeding (n = 47 pairs checked every ca. 5 hours 55–96 h after pairing), and allowed females to rear larvae until dispersal. We also maintained stock (or control) pairs where parents were allowed to rear their brood together until larval dispersal. We never saw males and females mating after the larvae had hatched in our experiments, so it is very unlikely that our experimental design is confounded by greater costs experienced by stock females associated with repeated mating. To address question 2) females were randomly assigned to one of three further care treatments. Widowed females were removed from breeding boxes around hatching (71 h after pairing), 8 hours after hatching (79 h) or 24 hours after hatching (95 h), and housed individually under standard conditions. In all treatments, we measured total mass and size of each brood at dispersal.

After larval dispersal, adults were removed from the breeding boxes and housed individually. Females were then given a new unrelated, randomly chosen partner (either a virgin or an experienced partner, depending upon availability in the colony) to start a second breeding bout 14 days after they were initially paired (see [18] for a similar experiment). Carcass mass at first or second breeding did not differ among treatment groups (univariate ANOVA, always P>0.11). All females reared their second brood with their partner present throughout. When the larvae dispersed, we again measured total mass and size of each brood. Females were then placed into individual boxes and kept under standard conditions until their time of death, which was recorded. In total, we used 108 (0 h postnatal care: n = 26; 8 h postnatal care: n = 21; 24 h postnatal care: n = 21; full-time postnatal care: n = 20; stock: n = 20) female experimental subjects bred originally from 69 different pairs (1.38±0.10 SE females per pair, range 1–3). We also used 108 (88 removed and 20 stock) males bred originally from 65 different pairs (1.41±0.10 SE males per pair, range 1–4).

### Statistical analyses

As data were not normally distributed, the effect of care treatment at first breeding (four-level fixed factor) on female lifespan (days since eclosion) and brood mass and size at dispersal was tested in mixed models assuming a Poisson error distribution. Because a number of our experimental subjects were raised by the same pair (see above), family of origin was entered as random intercept effect where necessary. However, the effect of partner removal (two-level fixed factor) on lifespan and subsequent reproduction of full-time widowed vs. stock females was tested among families that contributed to the analysis only one individual (n = 17 full-time and 13 stock females) because there were too few replicates per family to be able to incorporate a random intercept effect. Brood mass at dispersal was highly correlated with brood size (r = 0.958, P<0.001, n = 414 breeding events); we report only results involving brood mass at dispersal here. Carcass mass at first and second breeding and brood mass at dispersal from the second breeding attempt were entered as covariates when specified. Analyses were run with SAS 9.1. Degrees of freedom were estimated by the between-within variance partitioning method. Post-hoc comparisons were performed adopting Sidak correction.

## Results

### Effect of partner presence on maternal lifespan and subsequent reproduction

**Figure 1:**
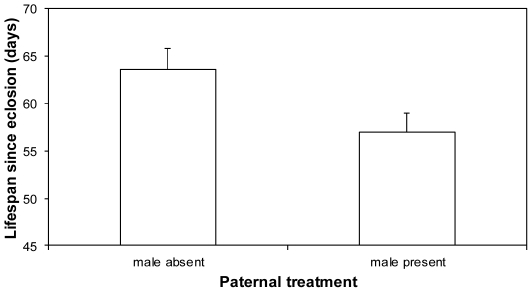
Mean (+SE) lifespan of females that cared for larvae until they dispersed, either when widowed before hatching or when partners were present throughout the first brood.

Surprisingly, females that reared their first brood with their partner had shorter lives than those that reared their first brood alone (F_1, 28_ = 5.53, P = 0.026; Figure 1). This result stood even after controlling for carcass mass at first or second breeding or brood mass at second breeding (always F_1, 27_>1.27, P>0.27 for these covariates). Furthermore, it could not be explained by differential maternal investment in the first brood according to the presence or absence of a partner, as during the first breeding round, brood mass at dispersal was similar whether the brood was reared by both parents (4.95 g±0.59 SE) or the female alone (4.27 g±0.63 SE; F_1, 28_ = 0.74, P = 0.40). Conversely, although the male's presence during postnatal care shortened the female's lifespan, we could detect no equivalent effect on the female's fecundity in her subsequent breeding attempt (F_1, 28_ = 1.99, P = 0.17).

### Effect of uniparental larval care on maternal lifespan and subsequent reproduction

**Figure 2:**
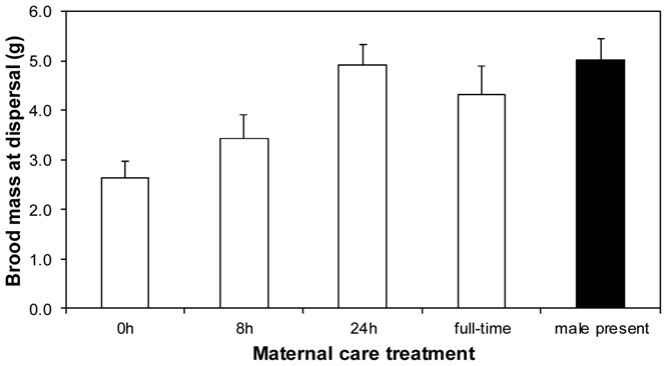
Mean (+SE) brood mass at dispersal at first breeding, in relation to the duration of maternal care.

Data collected during the first breeding round showed that the longer females spent with their offspring, the greater the level of care they provided, because larval mass at dispersal increased accordingly (F_3, 27_ = 5.76, P = 0.004; Figure 2). Broods where mothers were removed around hatching were significantly lighter than broods raised under the 24 h (P = 0.003) and full-time female-only care treatments (P = 0.038). Brood mass at dispersal was not related to initial carcass mass (F_1, 26_ = 0.03, P = 0.86).

**Figure 3:**
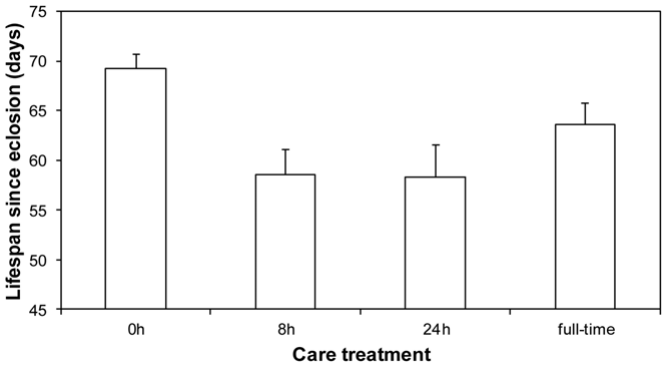
Mean (+SE) lifespan of experimentally widowed females in relation to the time spent caring for their first brood.

Females suffered costs of care in relation to the time spent with their offspring. Those removed from their first brood around hatching had a longer lifespan than females removed after 8 h and 24 h of larval care (F_3, 27_ = 4.80, P = 0.008, Figure 3; *post-hoc* comparisons always P<0.029). Contrary to our expectations, however, females removed at hatching did not live significantly longer than those that reared their offspring full-time until dispersal (P = 0.40). Family of origin also affected female lifespan (z = 3.23, P = 0.001). These results held when we controlled for carcass mass in the first or second breeding attempt (always P>0.52).

**Figure 4:**
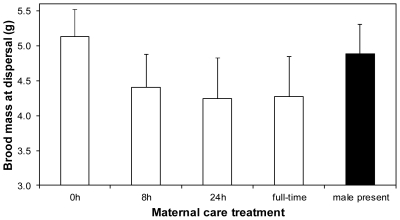
Mean (+SE) brood mass at dispersal at subsequent breeding bout, in relation to the duration of maternal care at first breeding.

Although the duration of maternal care in the first breeding attempt influenced female lifespan, we could detect no equivalent effect on female fecundity in the second breeding attempt (F_3, 27_ = 0.93, P = 0.44; Figure 4). However, independent of our experimental manipulation in the first breeding attempt, females that lived longer raised larger second broods than those that died sooner (F_1, 26_ = 6.80, P = 0.015; slope: 0.020±0.008 SE).

## Discussion

Contrary to expectation, our experiments revealed that females benefitted from experimentally simulated male desertion, subsequently living longer if they cared for offspring alone than if their partner was present until larval dispersal (Figure 1). How did this counter-intuitive result arise? Detailed analysis of the costs incurred at successive stages of larval care found that the costs of care were especially great in the hours immediately following larval hatching, when offspring were most dependent on their mothers for nourishment [14]. Nonetheless, females were able to recoup some of these costs if they then stayed with their offspring until dispersal. In fact, we could detect no difference in subsequent lifespan between females that were removed before providing any larval care at all and those who cared for larvae until dispersal (Figure 3). The most likely explanation is that females were able to feed from the carcass themselves (*e.g.* [20]–[22]) once their offspring had started self-feeding, gaining more from the unlimited access to higher quality nourishment available on the carcass than they could from food supplied under standard housing conditions when removed from the breeding box. In this way, we suggest, females were able to replenish reserves that were depleted by provisioning young in the first 24 hours after larval hatching. Perhaps the presence of males throughout a breeding attempt prevents females from feeding on the carcass themselves, or even results in competition for food, and this in turn prevents them from recouping the costs incurred during initial larval care. Consistent with this suggestion, the lifespan of mothers who raised offspring with a male (57.00±1.98 SE days) was very similar to the lifespan of mothers removed from the carcass 8 h to 24 h after hatching (58.36±2.06 SE days). Furthermore, observations of other *Nicrophorus* species suggest that females consume significant amounts of the carcass themselves during reproduction [20]–[22].

Although we found that the presence of the male throughout reproduction had a negative effect on female lifespan, we were unable to detect a corresponding effect on female fecundity in the subsequent breeding attempt. One possibility is that any such effect was too small to be detected with our sample size (Figure 4). Furthermore, in our previous work with similar sample sizes, fecundity costs associated with reproduction could only be detected from the third breeding attempt onwards [*e.g.* 11], [13], [19] whereas here we confined our attention only to the second breeding bout. It might be argued that we should not conflate effects of the male's presence on female longevity alone with effects on her fitness, because in many insects the two are not correlated. However, we think that the natural history of the burying beetle renders this objection invalid. Burying beetles are opportunistic breeders, dependent on the random appearance of a carcass to breed. Prolonging survival therefore potentially increases fecundity by increasing the likelihood of encountering another corpse. Indeed, our previous experimental work shows that burying beetles practice reproductive restraint, holding back resources from current reproduction so as to prolong lifespan, presumably to increase the chance of breeding again [19]. For this reason, lifespan is an important component of fitness in the burying beetle [11].

How do these results fit with our understanding of mate desertion as a manifestation of sexual conflict? Previous work shows that desertion by males at an early stage of reproduction can never be in the female's interests because she loses assistance in guarding the valuable carcass breeding resource [10], [12], and in preparing it for reproduction [13]. Here we focus on a later period, by examining the effects of male desertion on components of female fitness when carcass preparation is complete, and show that by this point desertion can be beneficial for females. Desertion by males increases female lifespan, thereby offering her the opportunity for a greater number of reproductive bouts [11], [13], so potentially increasing her lifetime reproductive success.

We can think of two possible co-evolutionary interpretations of our results, which remain to be tested in future work. One possibility is that our results constitute an example of so-called ‘cooperative adaptation’ (Figure 4b, scenario 4 in [2]). Here, females might have counter-adapted to mate desertion at an early stage of larval development [15]–[17], so that they now rely on the male's absence towards the end of the breeding bout as an opportunity to recoup the high costs of provisioning larvae incurred soon after hatching. According to this scenario, desertion by males is therefore now optimal for females as well as males. Alternatively, our results might be viewed as an instance of so-called ‘adaptation’ (see Figure 4b, scenario 5 in [2]), whereby females now prefer the male to leave sooner than is optimal for him. In other words, sexual conflict over mate desertion might now centre not on a race to leave first (*e.g.* [4]), but instead on a competition to stay the longest, to continue to exploit the resources available on the carcass. Consistent with this possibility, Bartlett observed that males deserted the brood much sooner when breeding on a very small carcass [10]. In three instances, males breeding on a small carcass were even killed by the female, presumably as females attempted to drive their partners away from the scant resources remaining on the carcass [10]. Future experiments that simulate male desertion at different times after hatching and measure the resulting payoffs for each sex may distinguish these two types of coevolutionary response. In the meantime, this study suggests that the coevolution of sexually antagonistic traits (in which adaptations in one sex drives counter-adaptations in the other) is not confined to mating behaviour, but includes sexual conflict over parental investment as well.
